# A novel approach to monitoring the efficacy of anti-tumor treatments in animal models: combining functional MRI and texture analysis

**DOI:** 10.1186/s12885-018-4684-z

**Published:** 2018-08-20

**Authors:** Ming Meng, Huadan Xue, Jing Lei, Qin Wang, Jingjuan Liu, Yuan Li, Ting Sun, Haiyan Xu, Zhengyu Jin

**Affiliations:** 1 Department of Radiology, Chinese Academy of Medical Sciences & Peking Union Medical College, Peking Union Medical College Hospital, No.1 Shuaifuyuan, Dongcheng District, Beijing, 100730 China; 2 Department of Biomedical Engineering, Chinese Academy of Medical Sciences & Peking Union Medical College, Institute of Basic Medical Sciences, No.5 Dongdan, Dongcheng District, Beijing, 100730 China

**Keywords:** Breast cancer, Neoadjuvant chemotherapy, Functional MRI, Texture analysis, Multiparameter imaging

## Abstract

**Background:**

The aim of this study was to evaluate the early anti-tumor efficiency of different therapeutic agents with a combination of multi-b-value DWI, DCE-MRI and texture analysis.

**Methods:**

Eighteen 4 T1 homograft tumor models were divided into control, paclitaxel monotherapy and paclitaxel and bevacizumab combination therapy groups (*n* = 6) that underwent multi-b-value DWI, DCE-MRI and texture analysis before and 15 days after treatment.

**Results:**

After treatment, the tumors in the control group were significantly larger than those in the combination group (*P* = 0.018). In multi-b-value DWI, the ADC_slow_ obviously increased in the combination group compared to that in the others (*P* < 0.01). The f increased in the control and paclitaxel groups, but the combination group showed a significant decrease versus the others (*P* < 0.02). Additionally, in DCE-MRI, the decreasing K^trans^ showed an evident difference between the combination and control groups (*P* = 0.003) due to the latter’s increasing K^trans^. The intra-group comparisons of tumor texture in pre-, mid- and post-treatments showed that the entropy had all significantly increased in all groups (*P* < 0.01, SSF = 0–6), though the MPP, mean and SD increased only in the combination group (P_MPP,mean,SD_ < 0.05, SSF = 4–6). Moreover, the inter-group comparisons revealed that the mean and MPP exhibited significant differences after treatment (P_mean,MP*P*_ < 0.05, SSF = 0–3).

**Conclusion:**

All these results suggest some strong correlations among DWI, DCE and texture analysis, which are beneficial for further study and clinical research.

## Background

Functional magnetic resonance imaging (fMRI) has grown very rapidly because it provides non-invasive and accurate imaging, especially its ability to discriminate tissue characteristics. Furthermore, using the characteristics of lesions, fMRI provides real-time and non-destructive measurements of pathological processes in vivo for early diagnosis and therapy evaluation. The two types of novel fMRI scanning techniques, multi-b-value diffusion-weighted imaging (DWI) and dynamic contrast-enhanced MRI (DCE-MRI), can potentially detect major diseases such as breast cancer. In general, DCE-MRI has shown high sensitivity in the detection of breast cancer (89–100%) and DWI has shown utility in predicting proper therapeutic regimens and monitoring responses to treatments [1]. Intra-tumoral vascular heterogeneity is essential for tumor treatments. Accordingly, antiangiogenic therapy is considered a highly promising new strategy to prevent tumor growth and metastasis. These two functional MRI techniques are able to measure the microvascular structure and reflect its permeability [2]. Several qualitative and semiquantitative parameters of DCE-MRI, ranging from simple semiquantitative inspection of the time-intensity curves to more sophisticated tracer kinetics modeling, can provide information on vascular permeability within the tumor [3]. Additionally, the values of apparent diffusion coefficient (ADC), which are based on the relative signal intensity change of the tumor tissue with increasing b values in multi-b-value DWI, can provide microstructural information at the cellular level. The changes in the ADC values correlated inversely with the tissue and cell densities [4, 5]. Therefore, these two imaging methods can potentially be used to monitor and evaluate the therapeutic effects of antiangiogenic therapy in the early stages of treatment.

Recent clinical studies show that bevacizumab, a genetically engineered humanized monoclonal antibody, is very efficient in curing various tumors because of its anti-VEGF activity. Bevacizumab can specifically combine with VEGF and impede the binding of VEGF to VEGFR to inhibit new vascular formation and suppress tumor growth with low toxicity [6]. As a control, another commonly used chemotherapeutic agent, paclitaxel, can bind to β-tubulin and stabilize the microtubules to restrain cell mitosis and inhibit cell proliferation [7]. As noted above, a promising approach would be to use multi-b-value DWI and DCE-MRI in combination to appraise the anti-angiogenic activity of bevacizumab compared with that of paclitaxel.

To ensure the accuracy of our research, we adopted an alternative new technique, texture analysis, to analyze and verify the imaging results. As a new imaging biomarker introduced in oncologic imaging, texture analysis can quantify the regional heterogeneity of a tumor, which is a recognized feature of malignancy and is associated with aggressive biology, inferior prognosis and treatment resistance [8]. Therefore, this image processing algorithm can be used to scan for subtle intra-tumoral anomalies by assessing the distribution of texture coarseness. The important texture parameters, including mean intensity, standard deviation of the gray-level histogram distribution, entropy (irregularity of gray-level distribution), skewness (asymmetry of the histogram), and kurtosis (flatness of the histogram) can reflect diverse information ranging from anatomical structure to biological function [9]. Previous studies have shown that compared to other imaging and biological parameters, coarse texture features may reflect the underlying vasculature as defined by CD34 [10]. According to this research, it is of value to perform texture analysis on the functional MRI findings and evaluate the correlation between the results.

## Methods

### Animal models

All animal experiments and relevant details were conducted in accordance with the approved guidelines and were approved by the committee on Animal Care and Use of Peking Union Medical College Hospital, Chinese Academy of Medical Sciences & Peking Union Medical College.

Balb/c-nu mice (female, 6 weeks old, approximately 20 g body weight) were purchased from the Beijing Vital River Laboratory Animal Technology Co., Ltd. (Beijing, China). The mice were maintained on sterilized food and water. The murine breast cancer cell line 4 T1 was obtained from the Cell Bank of the Chinese Academy of Science (Beijing, China) and maintained in Dulbecco’s minimum essential medium (DMEM) supplemented with 10% fetal bovine serum, penicillin (100 units/ml) and streptomycin (100 units/ml) and incubated at 37 °C in a 5% CO_2_ air environment. The breast tumors in the Balb/c-nu mice were established by subcutaneous inoculation with 3.5 × 10^6^ 4 T1 cells in 400 μl PBS.

### Treatment

The therapy was initiated after the tumors reached approximately 150 mm^3^ in volume. Then, these 4 T1 breast tumor homograft-bearing mice were randomized into three groups: control, paclitaxel monotherapy and combination therapy with antiangiogenic bevacizumab (Avastin, Roche, Switzerland) and paclitaxel. All of the mice were treated with intraperitoneal injections every three days. Sterile saline was used in the control group with a volume of 100 μl, and a dose of 10 mg/kg was used in the paclitaxel monotherapy group. In the combination therapy group, the mice were treated with the same dose of 10 mg/kg each [11]. The whole treatment process lasted for 15 days. This study included 18 mice carrying breast tumor homografts. All of the mice were scanned immediately prior to the treatment and 15 days after the initiation of the treatment. All the mice were sacrificed by cervical dislocation after the last scanning procedure. The tumor tissues from these three groups were subjected to histopathological analyses of vascularization.

### MRI protocol

All MRI examinations were performed on a GE Discovery MR750 3.0 T horizontal bore superconducting magnet coupled with a 35 mm diameter small animal coil (GE, Waukesha, USA). The animals were anesthetized by an intraperitoneal injection of 1% pentobarbital sodium with a volume of 150 μl. Heartbeats and respiration rates were monitored during the experimentation. The image acquisition included the routine T2WI, multi-b-value DWI and DCE-MRI. Multi-b-value DWI was acquired with 11-grade b values using a spin-echo sequence (0, 20, 50, 100, 200, 400, 600, 800, 1000, 1200, 1500 s/mm^2^, TR = 2500 ms, TE = 78 ms, FOV = 50 mm, matrix 64 × 64, slice thickness 1 mm, 11 slices). The DCE-MRI was followed by a 200-phase dynamic series of T1WI 2D FSPGR images with identical geometry and a temporal resolution of 3 s. To acquire a full range of images, all tumors were imaged with five coronal slices. Other DCE-MRI parameters were included as follows: TR = 9.7 ms, TE = 3.7 ms, FOV = 50 mm, matrix 192 × 96, flip angle 30°, slice thickness 2 mm. An intravenous bolus dose of 0.1 mmol/kg of Gd-DTPA was given after the 10th baseline data point through a catheterized tail vein tube.

The relevant parameters were measured after MRI examinations. ADC_slow_ (pure molecular diffusion), ADC_fast_ (perfusion-related diffusion), and f (perfusion fraction) were obtained from a bi-exponential IVIM model of multi-b-value DWI. Pharmacokinetic parameters of CER (contrast enhancement ratio), K^trans^ (transfer rate constant), K_ep_ (reverse rate constant), V_e_ (extravascular extracellular volume fraction), fPV (fraction of plasma volume) and AUC_90_ (area under curve 90 s) were obtained from a two-compartment model of DCE-MRI.

### Texture analysis

The texture parameters were obtained using the advanced research software algorithm TexRAD, an image-histogram technique invented at the University of Sussex (United Kingdom). From the axial T2 weighted images of all animals, the regions of interest (ROIs) were defined as the tumor outline in the largest cross-sectional images performed by an experienced radiologist (8 years of experience in imaging analysis) with manual delineation [12]. The ROI areas were selected with different spatial scale filter (SSF) values from 0 to 6 mm to extract MR texture features. SSFs of 0 and 2 reflect fine texture scales; SSFs of 3, 4, and 5 reflect medium texture scales; and an SSF of 6 reflects a coarse texture scale. The heterogeneity of these tissues was indicated by the following histogram parameters: mean intensity (the average value of all pixels in ROI), SD (the degree of dispersion between pixels and mean value in ROI. A high SD indicates that the data points are spread out over a large range of values.), entropy (irregularity of pixel intensity distribution in ROI), mean value of positive pixels (MPP, the average value of all the pixels that greater than zero), kurtosis (a measure of peakedness and tailedness of the histogram. The positive kurtosis means a histogram that is more peaked than a Gaussian (normal) distribution.), and skewness (a measure of asymmetry of the histogram. The positive skew means that the tail on the right side is longer than the left side, otherwise, the reverse.) [9, 13]. These quantitative parameters were associated with tumor histological features, such as blood and oxygen supply, necrosis, and fibrosis [14].

### Histopathology

All of the animals were euthanized after the last MRI examination. Then, the tumors were separated and the tissues were fixed by 10% formalin. Paraffin sections (2 mm thick) were acquired from the 4 T1 breast tumors. In addition, hematoxylin and eosin staining and immunohistochemical staining of CD31, CD34 and VEGF tests were performed to evaluate the neovasculature. The immunohistochemical staining was performed using rabbit anti-CD31 antibody (ab28364; Abcam, Cambridge, UK), rabbit anti-CD34 antibody (ab81289; Abcam) and rabbit anti-VEGF antibody (ab52917; Abcam). All the antibodies were diluted with tris buffered saline (TBS), which contains 1% bovine serum albumin (BSA). Based on these tests, the microvessel density (MVD) in these homografts was calculated.

### Statistical analysis

Quantitative parameters as described above were acquired from the functional MRI and analyzed in SPSS 20.0. The data under paclitaxel monotherapy and combination therapy were compared with the control condition by an analysis of variance. The correlations between MRI parameters and pathological features data were analyzed by linear regression.

Differences in the textural feature values before and after treatment within the control group, the paclitaxel monotherapy group and the combination therapy group were tested using the Mann-Whitney U test [15].

All of the tests were two-tailed. *P* values less than 0.05 were considered statistically significant.

## Results

### Tumor size measurements

**Fig. 1:**
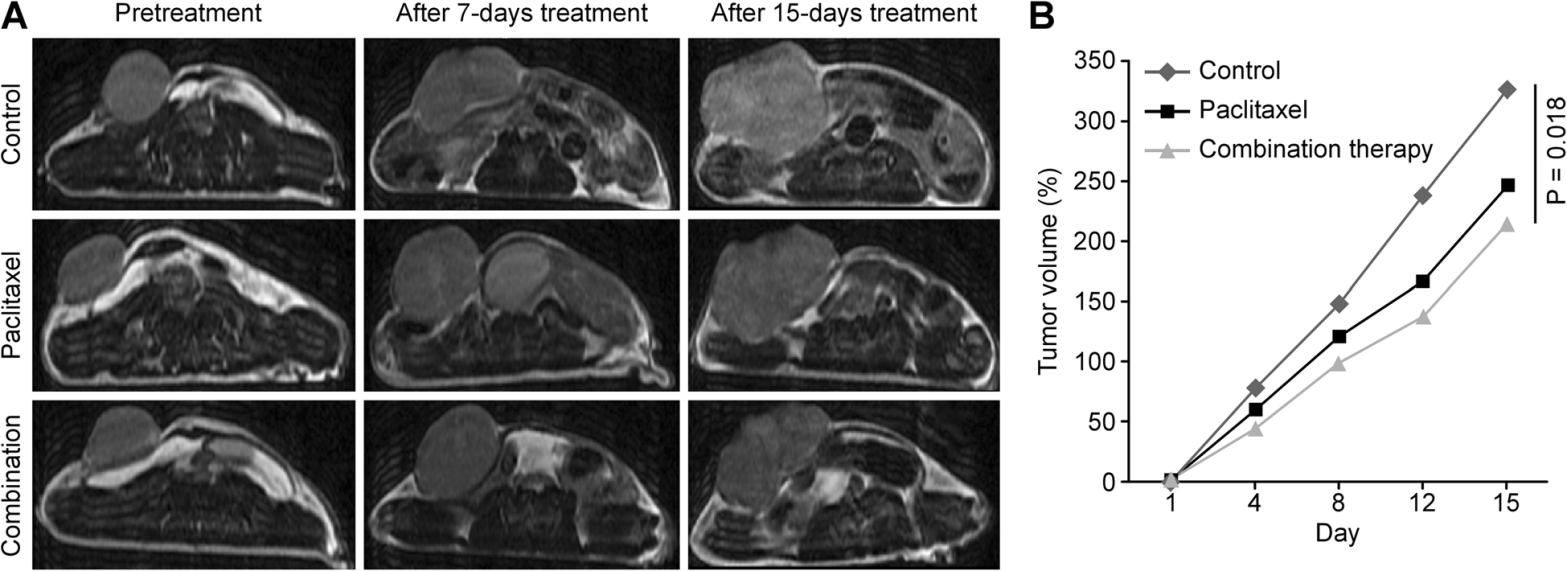
The tumor growth trends in the three groups. **a** The axial T2WI images of pre-treatment and after 7- and 15-day treatment with different therapies. The tumors became larger in the whole process and grew most quickly in the control group, which caused the surrounding organs to be constricted severely at the end of this trial. However, the combination group showed the slowest growth and the tumors remained relatively small and shallow in the late phases of treatment. The growth rate in the paclitaxel group was somewhere in the middle. **b** The percentage change of the tumor volume. The tumors exhibited nearly linear growth in the control group. There was no significant difference among the three groups on day 7 after therapy (*P* = 0.60). However, at the end of treatment, tumor growth was obviously suppressed by paclitaxel with bevacizumab combined therapy compared to the control group on day 15 (*P* = 0.018)

The baseline tumor volumes in the control, paclitaxel monotherapy and combination therapy groups were 192.4 ± 47.7 mm^3^, 263.7 ± 82.8 mm^3^ and 195.3 ± 85.2 mm^3^, respectively, with no significant differences (*P* = 0.26). Similarly, the growth of 4 T1-tumors in these three groups showed no conspicuous differences on day 7 after therapy (control, paclitaxel, paclitaxel with bevacizumab: 156.5 ± 48.7%, 119.3 ± 42.0% and 118.7 ± 48.0%, respectively; *P* = 0.60). However, after 15 days of therapy, the measurement results showed that tumors in the control group were significantly larger than in the combination therapy group. The tumor volumes reached 652.5 ± 142.8 mm^3^ with no therapy, and the tumor volumes reached only 416.2 ± 157.5 mm^3^ with paclitaxel and bevacizumab conjoint therapy (*P* = 0.018). The mean volume of the paclitaxel group was 521.2 ± 129.0 mm^3^. Accordingly, no obvious difference was found between the control and the paclitaxel monotherapy groups (*P* = 0.177) and the distinction between the two treatment groups was less intuitive (*P* = 0.055) (Fig. 1).

### DWI results

**Fig. 2:**
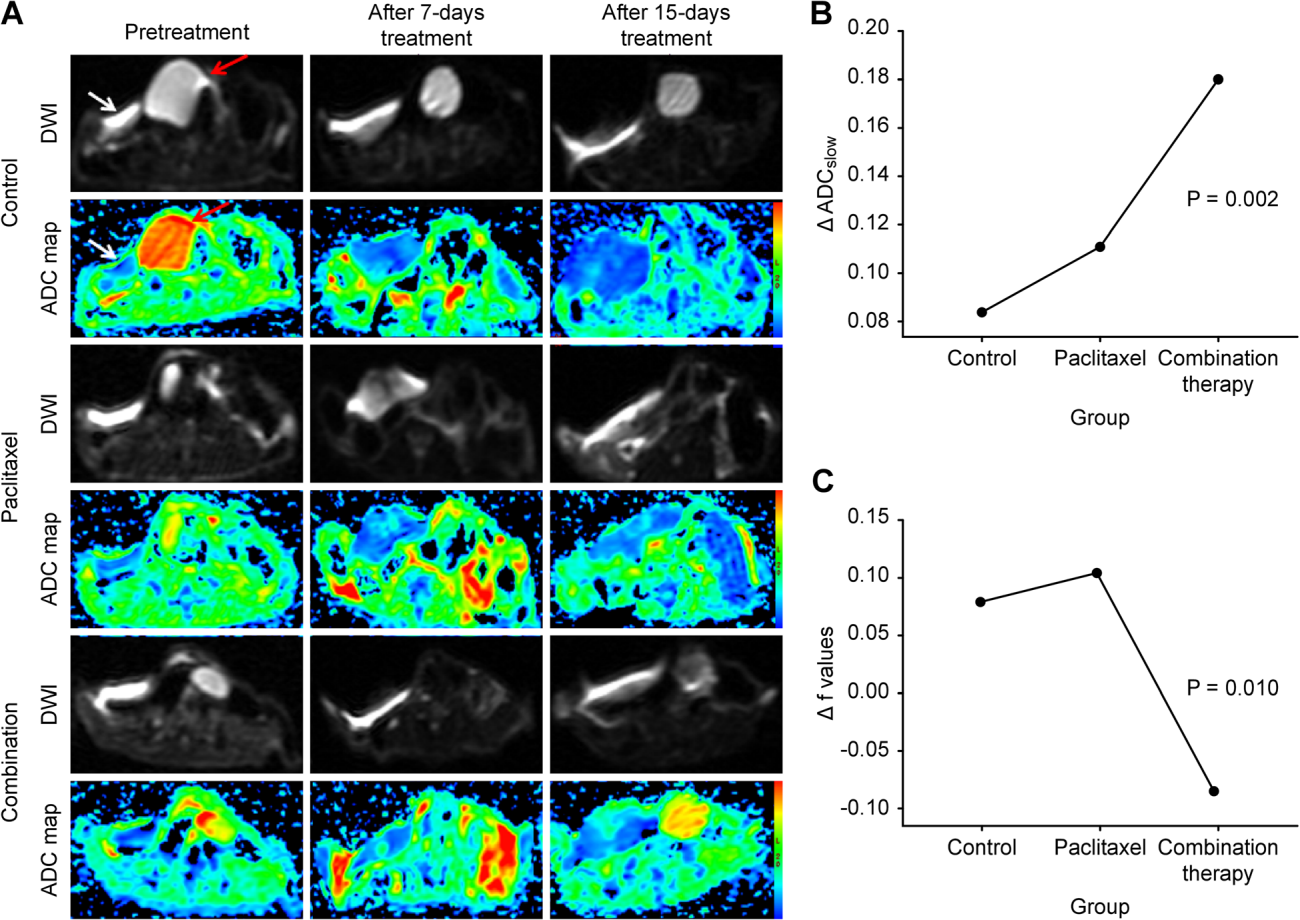
The multi-b-value DWI results in the three groups. **a** The DWI and ADC map of pre-treatment and after 7- and 15-day treatment with different therapies. The subcutaneous tumor (white arrow) was implanted near the bladder (red arrow). As seen from the ADC map, water molecular diffusion was much lower in the tumors (blue) than in the bladder (red). The tumor region always showed lower diffusion in the control group. However, after 7 days of anti-tumor therapies, the limitations of water diffusion improved in both the paclitaxel and the combination groups (the tumor central areas showed a slightly higher green signal). Furthermore, this improvement was more obvious in the combination group after 15 days of treatment. Meanwhile, marked diversities were observed in ADCslow (**b**) and perfusion fraction (f) (**c**) among the three groups before and after treatment. The changing tendencies were derived from ANOVA, which reflected the variations after 15 days of treatment according to their own separate patterns

The multi-b-value diffusion-weighted imaging (DWI) after all the treatments showed increasing trends of the ADC_slow_ value in these three groups, especially a distinct increase in the combination therapy group (control: 42.17 ± 19.0%, paclitaxel: 53.74 ± 24.16%, combined treatment group: 118.84 ± 47.59%, *P* = 0.002). There was a significant difference between the control and the combination treatment groups (*P* = 0.001), and the same difference was reflected in the two therapeutic groups (*P* = 0.008). Regrettably, no conspicuous difference was found between the control and the paclitaxel monotherapy groups (*P* = 0.269). Even more remarkably, the perfusion fraction (f) values showed the opposite behavior. Growth trends in f values were observed in the control and paclitaxel groups (control: 36.72 ± 17.47%; paclitaxel: 52.24 ± 36.35%), but the bevacizumab and paclitaxel combination group showed a decrease (− 25.12 ± 47.39%) on day 15 after the initiation of therapy. These variable trends caused remarkable distinctions among the three groups (*P* = 0.010). Meanwhile, the statistical differences between the control and combination therapy groups, as well as between the two therapeutic groups, were highly significant (*P* = 0.013, *P* = 0.005, respectively). There was no significant difference in the f values between the control and the paclitaxel monotherapy groups (*P* = 0.671) (Fig. 2).

### DCE-MRI results

**Fig. 3:**
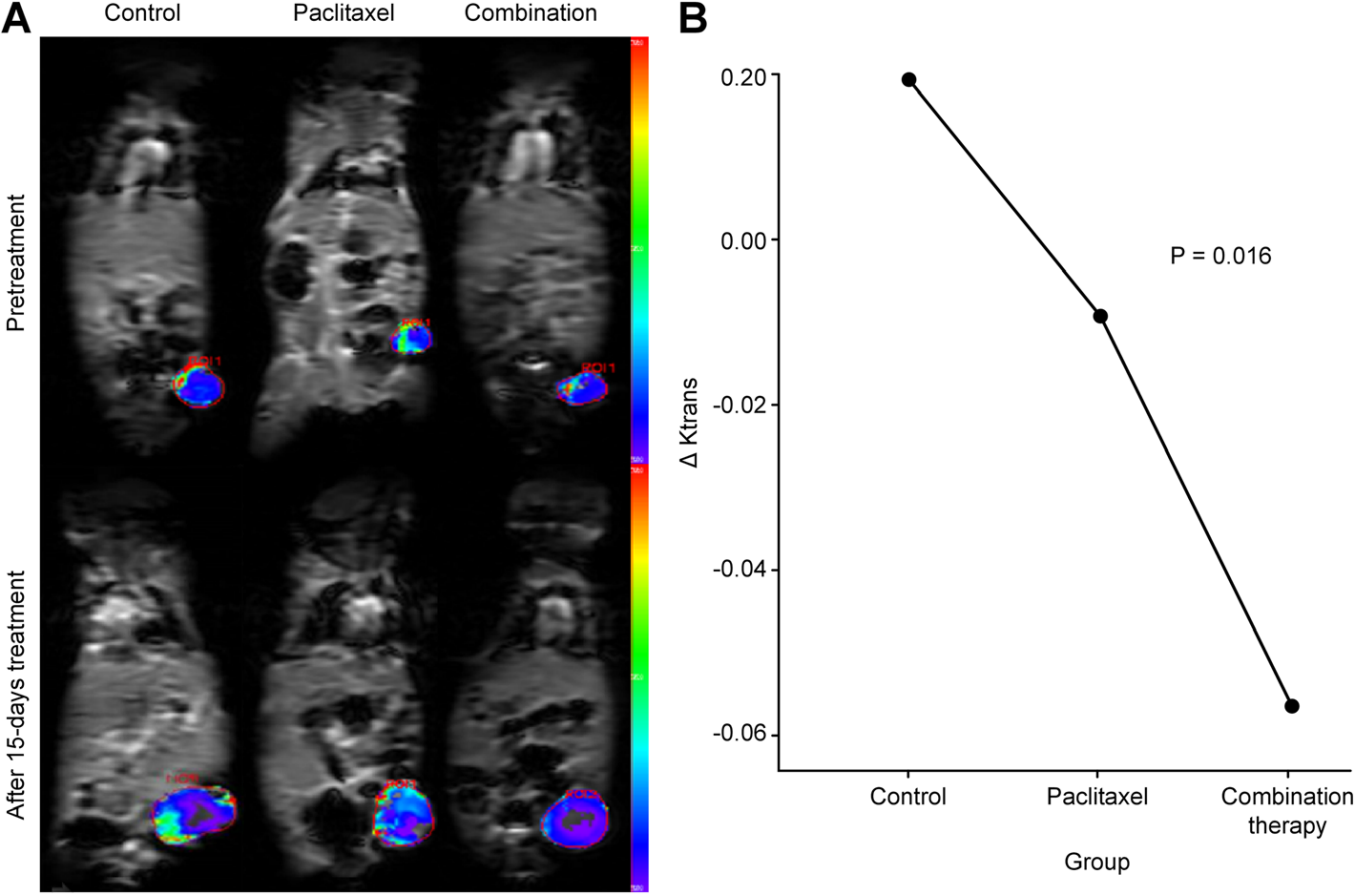
The DCE-MRI results in the three groups. **a** The K^trans^ maps derived from DCE-MRI on pre-treatment and after 15-day treatment in the three groups. As shown in the pictures, the blood supplies of the tumor margins were more abundant (red/green) than the central parts (blue) before treatment. Nevertheless, some differences emerged over 15 days of handling. The blood supply was more adequate in the control group, and the other two groups appeared to have nearly opposite distribution tendencies, especially the combination group. The quantitative analysis results further confirmed these changes and showed striking differences in K^trans^**b** among the three groups before and after treatment. The changing tendencies were also derived from ANOVA

A comparative analysis of the DCE-MRI results before and after anti-tumor therapy in the three groups exhibited significant differences. The transfer rate constant (K^trans^) values in the two therapeutic groups showed a significant decrease, but the control group showed an increase (paclitaxel:-28.8 ± 20.3%; combined treatment group: − 55.42 ± 30.43%; control: 127.37 ± 76.7%; *P* = 0.016) on day 15 after treatment. Accordingly, the statistical results were very similar to the DWI findings. There were significant differences between the control and combination treatment groups (*P* = 0.003) or between the two therapeutic groups (*P* = 0.044). No significant difference was detected in the K^trans^ values between the control and the paclitaxel monotherapy groups (*P* = 0.219). Furthermore, there were no significant differences in the other parameters among the three groups (Fig. 3).

### Texture analysis results

The analysis of tumor texture in pre-, mid- and post-treatment in these three groups to examine microstructural changes and therapy response revealed that the entropy values were continuously increasing with or without therapy in the three groups and that all the changes had statistical significance within the groups (*P* < 0.01 under all the SSF values from 0 to 6 mm). In addition, the MPP, mean intensity and SD values showed the same increasing tendency only in the combination therapy group for medium and coarse features (SSF = 4, 5, 6). These differences were statistically significant (*P*_*MPP*_ < 0.05, *P*_*mean*_ < 0.05, *P*_*SD*_ < 0.03, respectively) (Table 1).

**Table 1:** The intra-group comparisons of texture parameters in the control, paclitaxel and combination therapy groups

SSF	Pre-treatment				Mid-treatment				Post-treatment				*P* value			
	mean	SD	entropy	MPP	mean	SD	entropy	MPP	mean	SD	entropy	MPP	mean	SD	entropy	MPP
A. Texture parameters in the control group																
0	1725.70 ± 139.32	289.54 ± 24.82	6.12 ± 0.18	1725.70 ± 139.32	1599.25 ± 90.58	311.06 ± 29.68	6.55 ± 0.07	1599.25 ± 90.58	1658.22 ± 148.93	341.60 ± 39.17	6.74 ± 0.05	1658.22 ± 148.93	0.330	0.105	0.002*	0.330
2	1195.15 ± 808.31	1056.11 ± 345.60	6.48 ± 0.32	1579.19 ± 425.70	817.32 ± 638.70	1138.52 ± 313.80	7.22 ± 0.11	1373.25 ± 305.27	1351.10 ± 94.28	1028.40 ± 141.74	7.53 ± 0.10	1537.98 ± 79.00	0.651	0.733	0.002*	0.677
3	2075.70 ± 815.16	1379.17 ± 426.13	6.53 ± 0.30	2266.05 ± 692.00	1331.32 ± 712.57	1111.32 ± 122.70	7.21 ± 0.08	1649.98 ± 518.25	1955.66 ± 266.91	962.22 ± 103.47	7.49 ± 0.13	1997.08 ± 237.50	0.196	0.031*	0.002*	0.196
4	2589.17 ± 737.94	1449.94 ± 405.76	6.52 ± 0.32	2758.20 ± 605.26	1816.10 ± 679.22	1243.09 ± 352.48	7.22 ± 0.07	2121.33 ± 504.72	2524.37 ± 430.15	987.92 ± 144.55	7.51 ± 0.12	2533.12 ± 419.03	0.228	0.179	0.002*	0.230
5	2982.66 ± 619.22	1321.09 ± 420.85	6.51 ± 0.31	3037.58 ± 567.00	2304.45 ± 604.56	1317.59 ± 488.49	7.23 ± 0.12	2556.24 ± 476.15	3032.57 ± 522.03	1072.23 ± 139.86	7.51 ± 0.15	3032.57 ± 522.03	0.141	0.403	0.003*	0.185
6	3390.92 ± 537.48	1161.66 ± 396.02	6.50 ± 0.31	3390.92 ± 537.48	2816.54 ± 544.16	1289.99 ± 452.01	7.21 ± 0.13	2883.07 ± 518.08	3444.29 ± 569.76	1108.20 ± 113.03	7.56 ± 0.11	3444.29 ± 569.76	0.134	0.733	0.002*	0.164
B. Texture parameters in the paclitaxel group																
0	1792.59 ± 269.74	294.36 ± 31.16	6.20 ± 0.07	1792.59 ± 269.74	1720.35 ± 154.36	319.42 ± 95.00	6.57 ± 0.17	1720.35 ± 154.36	1723.30 ± 132.64	343.89 ± 33.77	6.71 ± 0.08	1723.30 ± 132.64	0.970	0.185	0.005*	0.970
2	1506.90 ± 971.82	1098.25 ± 196.70	6.65 ± 0.16	1775.18 ± 729.63	1425.57 ± 422.54	1195.67 ± 413.08	7.34 ± 0.26	1799.71 ± 207.05	1732.09 ± 387.71	1072.68 ± 138.56	7.55 ± 0.26	1867.67 ± 304.82	0.595	0.932	0.008*	0.970
3	2180.45 ± 944.57	1243.56 ± 484.91	6.65 ± 0.21	2413.68 ± 750.85	2140.12 ± 281.63	1116.57 ± 234.65	7.32 ± 0.25	2226.54 ± 155.82	2542.73 ± 582.90	1054.99 ± 156.52	7.52 ± 0.22	2557.25 ± 575.12	0.619	0.914	0.006*	0.914
4	2703.43 ± 918.57	1423.35 ± 694.75	6.66 ± 0.24	2862.68 ± 929.52	2668.49 ± 298.86	1093.71 ± 243.84	7.31 ± 0.24	2681.39 ± 299.52	3136.81 ± 686.17	1184.75 ± 233.59	7.56 ± 0.23	3145.29 ± 677.08	0.595	0.651	0.005*	0.566
5	3076.44 ± 1148.06	1401.55 ± 733.68	6.65 ± 0.21	3157.93 ± 794.39	3063.26 ± 529.59	1103.85 ± 390.31	7.28 ± 0.30	3075.22 ± 541.38	3509.64 ± 706.55	1268.90 ± 275.16	7.59 ± 0.26	3513.13 ± 703.86	0.566	0.691	0.005*	0.482
6	3383.79 ± 901.65	1256.03 ± 624.43	6.64 ± 0.21	3405.85 ± 715.29	3396.54 ± 717.25	1057.56 ± 423.29	7.27 ± 0.31	3396.54 ± 717.25	3718.2 ± 673.68	1204.06 ± 274.35	7.58 ± 0.29	3718.2 ± 673.68	0.691	0.878	0.005*	0.691
C. Texture parameters in the combination therapy group																
0	1806.38 ± 205.10	312.10 ± 66.52	6.10 ± 0.20	1806.38 ± 205.10	1678.27 ± 95.64	312.68 ± 59.26	6.43 ± 0.15	1678.27 ± 95.64	1884.06 ± 121.01	375.78 ± 41.57	6.68 ± 0.11	1884.06 ± 121.01	0.055	0.143	0.001*	0.055
2	1433.12 ± 720.31	1311.13 ± 379.57	6.43 ± 0.24	1894.66 ± 453.57	1854.46 ± 321.90	955.21 ± 131.04	7.07 ± 0.14	1927.35 ± 338.02	1910.08 ± 315.32	1212.64 ± 200.58	7.45 ± 0.15	2081.77 ± 351.62	0.368	0.064	0.001*	0.630
3	2448.26 ± 419.37	1599.97 ± 672.77	6.46 ± 0.26	2821.10 ± 506.02	2488.27 ± 377.97	1012.94 ± 139.51	7.09 ± 0.12	2504.52 ± 373.95	2791.28 ± 384.04	1334.57 ± 134.55	7.48 ± 0.16	2893.49 ± 361.47	0.318	0.030*	0.001*	0.164
4	3011.07 ± 359.31	1471.26 ± 652.16	6.42 ± 0.29	3154.51 ± 477.70	2785.42 ± 425.84	892.06 ± 122.07	7.04 ± 0.13	2788.44 ± 422.44	3433.14 ± 429.29	1308.58 ± 164.02	7.46 ± 0.16	3446.67 ± 417.53	0.029*	0.019*	0.001*	0.050
5	3355.47 ± 524.72	1295.85 ± 608.95	6.40 ± 0.32	3395.32 ± 561.31	2965.52 ± 528.24	811.48 ± 135.21	6.97 ± 0.16	2965.52 ± 528.24	3867.23 ± 421.55	1251.75 ± 188.81	7.43 ± 0.17	3867.23 ± 421.55	0.025*	0.023*	0.001*	0.034*
6	3678.70 ± 683.91	1115.19 ± 538.41	6.40 ± 0.29	3680.00 ± 685.25	3136.03 ± 593.59	763.13 ± 151.67	6.99 ± 0.19	3136.03 ± 593.58	4154.52 ± 361.67	1163.57 ± 163.15	7.42 ± 0.17	4154.52 ± 361.67	0.045*	0.024*	0.001*	0.045*

“*“means *P* < 0.05

There were no differences in the mean, SD, entropy or MPP among the three groups before treatment. With the implementation of various handling measures, compared to pre-therapy, the mean and the MPP values under fine and medium features using SSFs of 0, 2 and 3 mm demonstrated significant differences among the different groups at post-treatment (*P*_*mean*_ < 0.05 and *P*_*MPP*_ < 0.05). However, changes in the other parameters were not remarkable (Table 2).

**Table 2:** Comparisons among the three groups pre-, mid- and post-treatment

SSF	Pre-treatment (*P* value)				Mid-treatment (*P* value)				Post-treatment (*P* value)			
	mean	SD	entropy	MPP	mean	SD	entropy	MPP	mean	SD	entropy	MPP
0	0.892	0.283	0.524	0.892	0.326	0.817	0.419	0.326	0.049*	0.315	0.389	0.049*
2	0.863	0.526	0.336	0.574	0.031*	0.651	0.110	0.049*	0.110	0.264	0.263	0.041*
3	0.673	0.724	0.518	0.342	0.026*	0.649	0.099	0.043*	0.056	0.068	0.925	0.049*
4	0.621	0.975	0.328	0.574	0.060	0.124	0.057	0.080	0.056	0.077	0.473	0.056
5	0.692	0.975	0.369	0.557	0.127	0.199	0.065	0.194	0.098	0.182	0.480	0.098
6	0.557	0.924	0.357	0.557	0.326	0.173	0.131	0.392	0.338	0.422	0.235	0.338

“*“means *P* < 0.05

### Immunohistochemistry results

The histological analysis of the 4 T1 allograft tumors showed that the combined treatment caused significant tumor suppression and CD31 immunostaining had a higher specificity for new vessels than CD34. The quantitative analysis of microvessel density (MVD) was assessed by CD31 and revealed an obvious decrease in the combination therapy group after 15 days of treatment, which was in sharp contrast to the other two groups (combined treat group: − 17.61 ± 23.16% vs. control: 31.39 ± 30.41% vs. paclitaxel: 30.12 ± 27.65%). These detection results also had significant statistical differences (combination therapy vs. control/paclitaxel: *P* = 0.007/*P* = 0.006). Moreover, the same changing trends in MVD in the control and paclitaxel monotherapy groups did not cause significant differences (*P* = 0.907).

**Fig. 4:**
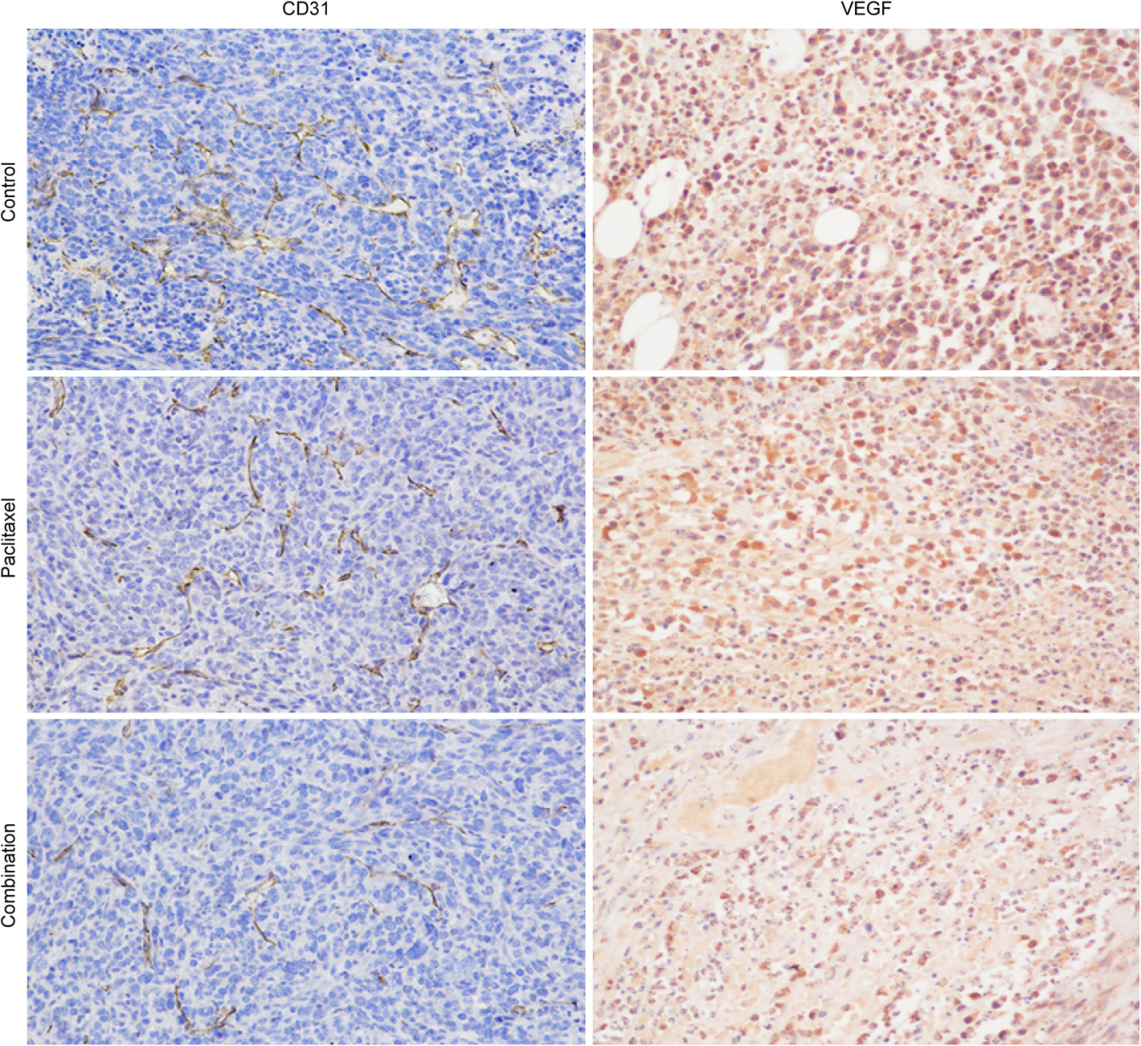
Immunohistochemical results (× 200) with CD31 and VEGF stains of control, paclitaxel- and combination-treated tumors after 15 days of treatment. The target substances were dyed brownish yellow. Both microvessel density (MVD) assessed by CD31 and the optical density of VEGF were obviously lower in the combination therapy group than in the other two groups

The average optical density of VEGF also showed the same changes among these groups. Through the combined treatment with bevacizumab and paclitaxel, the VEGF average optical density decreased (− 13.50 ± 57.25%), but the control and paclitaxel monotherapy groups exhibited increases (14.20 ± 44.41%, 27.50 ± 96.19%, respectively) (Fig. 4).

### Correlation analysis results

**Fig. 5:**
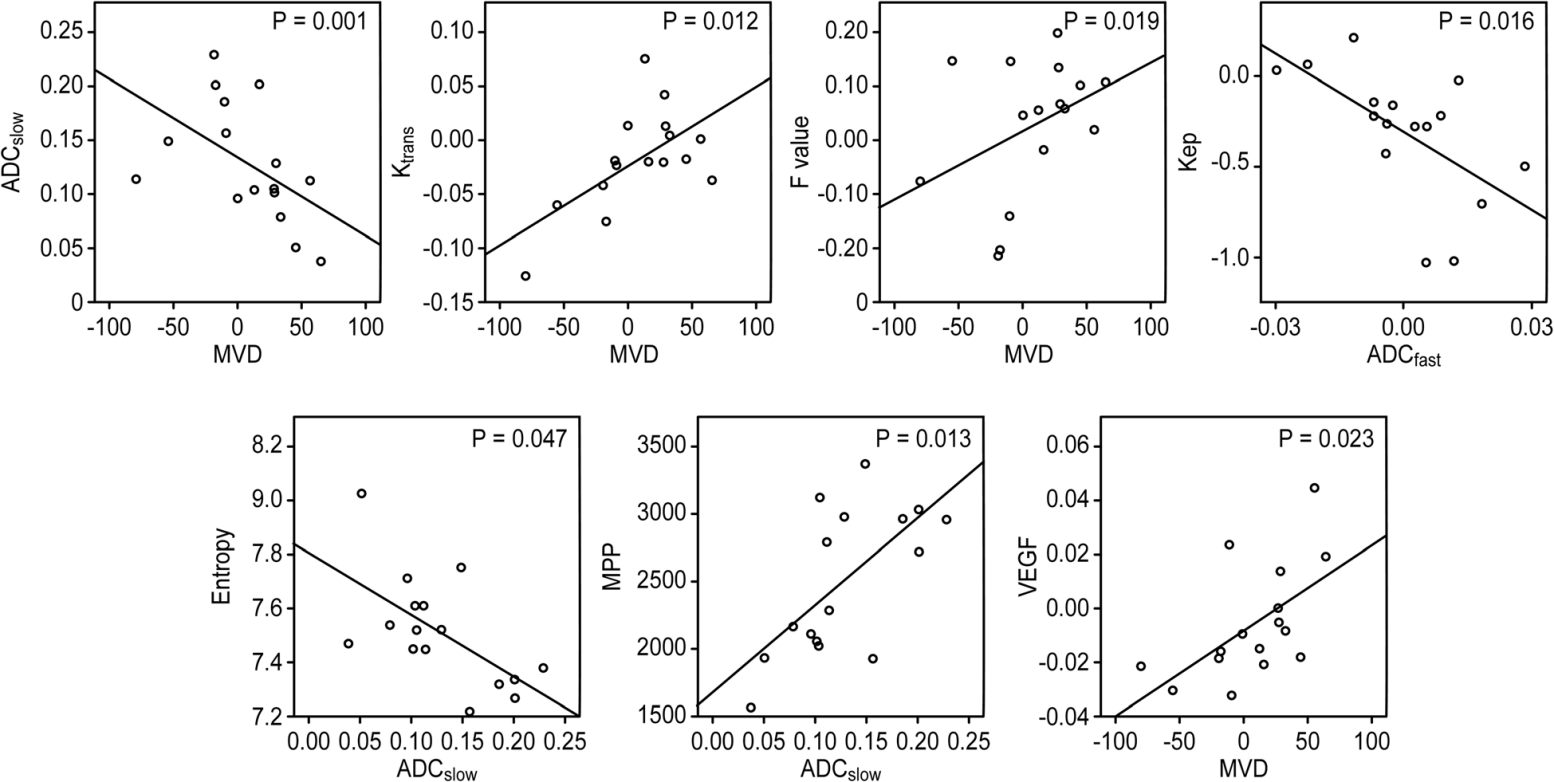
These linear maps can be used to directly reflect the correlation between the various parameters. Significant positive and linear correlations existed between MVD vs. K^trans^, perfusion fraction (f) and VEGF. However, MVD and ADC_slow_ were negatively correlated. In addition, ADC_slow_ values were significantly negatively correlated with entropy but positively correlated with MPP. There was also a strong correlation between the radiographic parameters of multi-b-value DWI and DCE-MRI, such as the inverse relationship between ADC_fast_ and K_ep_

To further clarify our research, an association study was performed with the above results. This analysis involved comparisons of MVD versus DWI/DCE-MRI, DWI versus DCE-MRI, and texture analysis versus DWI/DCE-MRI. The correlation coefficient ‘r’ of the percentage change of MVD versus K^trans^ was 0.612 (*P* = 0.012), that of MVD versus ADC_slow_ was − 0.810 (*P* = 0.001), that of MVD versus perfusion fraction (f) was 0.580 (*P* = 0.019), that of K_ep_ versus ADC_fast_ was − 0.593 (*P* = 0.016), that of ADC_slow_ versus entropy was − 0.503 (*P* = 0.047), and that of ADC_slow_ versus MPP was 0.603 (*P* = 0.013). In addition, MVD was positively correlated with the expression of VEGF (*r* = 0.563, *P* = 0.023) (Fig. 5).

## Discussion

In this study, we aimed to develop a practical approach to assessing the efficacy of early anti-tumor therapy. Previous studies have shown that angiogenesis can provide nutrition and oxygen to the tumor and thus plays a vital role in tumor progression [16]. Tumors grow exponentially when there is a blood vessel involvement, but they grow slowly and linearly in an avascular environment [17]. Therefore, anti-angiogenesis has an irreplaceable function in oncotherapy, and the antineoplastic agents that target tumor angiogenesis have become a hot research topic in recent years. As the first drug to be approved by the FDA to inhibit tumor angiogenesis, bevacizumab is well known for its high affinity in blocking angiogenesis induced by VEGF, which can induce the proliferation and migration of endothelial cells and increase the permeability of the microvasculature [18]. Normally, the gold standard for evaluating whether a drug is successful in inhibiting tumor angiogenesis is the MVD count. However, it is almost impossible to continuously remove tumor tissue from patients to observe the real-time efficacy of anti-angiogenesis therapy by calculating the microvessel density in clinical practice. It is encouraging that our study confirms that this problem can be solved by a new multi-parameter fusion analysis.

In this preclinical study, we found that many important imaging parameters were sensitive to different treatments. After the addition of bevacizumab, the changes in functional MRI and the texture analysis in the combination therapy group were very significant and caused a difference in tumor volume compared to that in the other groups. DWI has great advantages in reflecting the microstructure of tissues (high b-value) and the blood perfusion status (low b-value), especially its crucial parameter ADC [19, 20]. Therefore, if a treatment works, the cellular integrity will be disrupted, then the ADC_slow_ value, drawn from high b-value DWI, will rise due to the enhancement of water diffusion [21], which is supported by our research findings. With the occurrence of necrosis in tumor central positions, the ADC_slow_ value slightly increased without any therapy in the control group. However, when angiogenesis is blocked by bevacizumab, the nutrients needed for tumor growth would be insufficient and the resulting decrease in cell density would lead to a substantial increase in ADC_slow_ values. At the same time, the experimental data show that the inhibition of cell mitosis by paclitaxel induced cell density reductions that were inferior to bevacizumab, but the increase in ADC_slow_ was similar to the control group. Additionally, the f value assessed blood perfusion directly and showed significant differences in low b-value DWI between the groups. The results contrasted with ADC_slow_ and antiangiogenic therapy resulted in a significant decrease in the f value 15 days after therapy initiation, but the other two groups showed an opposite trend. Moreover, the changes in the f value exhibited a close association with MVD, but the changes in ADC_slow_ were strongly negatively correlated with microvessel counts. The very meaningful relevance of DWI parameters and histological results are fully consistent with earlier studies showing that DWI can be used to monitor the early therapeutic effects of vascular targeting agents [22].

DCE-MRI is the most common technique for non-invasive evaluations of tissue blood perfusion and is a valid method for monitoring the effectiveness of a variety of treatments by tracking the pharmacokinetics of Gd-DTPA [23]. The most commonly used parameter to reflect the vascular permeability and the blood flow rate and volume is K^trans^. Combined with other parameters, such as K_ep_, K^trans^ can reflect the degree of angiogenesis in tumors to a certain extent [24, 25]. Our study showed that high K^trans^ values appeared with the growth of tumors in the control group. This finding is diametrically opposite to the growth situation in the two therapy groups as the K^trans^ values were constantly dropping. The increase in K^trans^ values indicated increases in tumor blood perfusion and high capillary permeability that provided more nutrients for tumor growth and ultimately accelerated the proliferation of tumor cells. During the late phase of the experiment, the subcutaneous tumor volumes in the control group were significantly larger than in the other two groups, providing good verification for K^trans^. Additionally, the significantly different downward trends in the two therapy groups were caused by the different mechanisms of paclitaxel and bevacizumab. Paclitaxel has a definite anti-tumor effect by inhibiting the microtubule system. However, some scholars have confirmed that bevacizumab can improve the delivery and efficacy of paclitaxel [26]. The suppression of angiogenesis and vascular permeability by bevacizumab ensures the concentration of paclitaxel. The significant changes in volume, K^trans^ and other imaging parameters in the combination group compared to those in the paclitaxel-alone group and the control group likely occurred because the duration of therapy was not long enough to cause an obvious difference between the paclitaxel monotherapy and the control groups. Encouragingly, the histological results were consistent with DCE-MRI. The MVD counts showed a strong positive correlation with K^trans^. Through treatment with bevacizumab, the expression of VEGF in the combination group was reduced. In recent years, increasing attention has been given to K_ep_, and previous studies have shown that a high baseline value of K_ep_ corresponds to a high exchange fraction of a drug between the plasma and the extravascular extracellular space (EES), indicating potentially superior therapy efficacy [27]. Most likely, the individual differences, tumor cell necrosis, and other factors caused the contrast agent residue in the interstitial space and led to the error in extravascular extracellular osmotic volume, eventually causing the lack of significant changes in K_ep_ in our study. On the other hand, K_ep_ is also significantly affected by V_e_, which may be determined by cell density, cystic degeneration and tissue reaction, etc. According to Tofts [28], V_e_ is not a quite stable factor, because it’s often affected by the edema surrounding the lesion. Nevertheless, when we analyzed the correlation between DCE-MRI and DWI, we found that the K_ep_ was negatively related to ADC_fast_, which was drawn from low b-value DWI. Because the ADC value in the Double Exponential Model mainly reflects the tumor density characteristics, the increase in tumor density will certainly affect the contrast agent rate of return to the plasma from the EES. Therefore, it can be concluded from the above analysis that multi-b-value DWI and DCE are complementary to each other in the assessment of angiogenic function and tumor perfusion.

Although multi-b-value DWI and DCE-MRI have provided considerable information for monitoring tumor growth and oncological therapy efficacy, these two imaging techniques can be affected by many factors, such as the inhomogeneity of the tumor tissues, artifacts resulting from the subcutaneous tumor model and motion of the animal during the imaging process [29]. Additionally, the clinical images have some limitations in reflecting the cellular and molecular characteristics of lesions, such as cell proliferation and metabolism, necrosis and hypoxia [30]. Recently, a growing number of studies have attempted to clarify the measurement of heterogeneity in medical images by textural analysis, a second-order statistical technique with parameters derived from the distributions of local features, which may allow better tissue characterization, image segmentation, and prediction of therapy response and survival [31, 32]. Therefore, the major advantage of this potential tool is that it can maximize the information from clinical images without the need for additional acquisitions [9]. This advantage must be fully exploited in our research. By measuring the unenhanced T2-weighted MRI, we found that all of the allograft tumor-bearing mice were in the same condition before treatment, but with treatment and various handling, the entropy values increased significantly in the three groups under all SSFs. Entropy represents the disorder degree of the pixels in ROI, the higher its value is, the more is the disorder of tissue. A previous publication showed the severity is associated with the degree of texture coarseness which was correlated with glucose uptake measures (obtained from FDG-PET, *r* = 0.51, *P* = 0.03) [33]. It is therefore clear that the increasing glucose metabolism allowed the growth rate of this 4 T1 allograft tumor to increase, which was consistent with the increasing size of the tumors in all of the mice. According to Ng et al. [34], the heterogeneity of tumor tissues increased with growth. According to Ganeshan et al. [10] and Henriksson et al. [30], the increased image heterogeneity within tumors may be associated with differences in regional tumor cellularity, proliferation, hypoxia, angiogenesis and necrosis. Therefore, through the effects of anti-angiogenesis and inhibition of cell mitosis by combined therapy with bevacizumab and paclitaxel, the microstructures of tumor, including cells, extracellular matrix and microvasculature, would be disturbed, generating a series of variations on cellular and molecular levels that are too subtle to detect using traditional imaging diagnostic techniques. The persistent variations ultimately led to significant differences in the average value of the pixels within the lesions (mean intensity, *P* < 0.05) and high dispersion exists around the mean value (SD, *P* < 0.03). Because of the absence of strong and effective chemotherapy, obvious changes did not appear in the other two groups after treatment. In a nuclear medicine study, the scholars found that tumors with more heterogeneous water distribution (i.e., higher SD and mean value of positive pixels, MPP) were more glycolytic [35]. This conclusion was also supported by our empirical evidence. When angiogenesis was blocked by bevacizumab, the reduction in tissue perfusion limited the oxygen supply to the tumor, which led to significant dependence on energy from glycolysis compared with before treatment (P_MP*P*_ < 0.05). Another finding that supports this statement is that the changes in the mean, SD and MPP all occurred in medium and coarse texture scales, which were more inclined to reflect biological characteristics as genomics analyses based on the investigation by Chowdhury et al. [35]. Furthermore, the above analyses were applicable to the comparison among the different groups. The discrepancies on cellular and molecular levels, such as anti-proliferation, hypoxia, angiogenesis and necrosis induced by monotherapy and combination therapy, eventually caused the diversities in anatomical structure (under fine and medium texture scales) that embodied the dramatic differences in both the average value of the pixels (mean, *P* < 0.05) and the positive pixels (MPP, *P* < 0.05) within the tumor region. These major structural changes could be observed in traditional imaging parameters, as described above. As in our study, textural analysis was not independent; it was closely related to functional magnetic resonance imaging. Entropy was significantly negatively correlated with ADC_slow_ (*r* = − 0.503, *P* < 0.05). A higher entropy represents increased heterogeneity, which signifies the restriction of water diffusion (lower apparent diffusion coefficient) to some extent. Surprisingly, the increasing MPP value was remarkably positively correlated with ADC_slow_ (*r* = 0.603, *P* = 0.013), probably because the more glycolytic environment (higher MPP) produced metabolites that increased the permeability of the cell membrane and facilitated the diffusion of water molecules. However, further confirmation is warranted.

Admittedly, there are several limitations in our study. The vulnerability of six-week-old nude mice and other factors led to high mortality during the experiment; thus, the animal tumor model was achieved in a limited number of mice. In addition, the susceptibility artifacts in DWI at air-soft tissue borders in the subcutaneous tumor model [29], the motion of animals during the imaging process, and the fact that implanted tumors are more homogeneous than primary tumors caused inevitable system errors. In further studies, we will strive to overcome these limitations and explore more diverse, multimodality fusion imaging methods.

## Conclusion

This study shows successful monitoring of the early phases of antiangiogenic therapy using multi-b-value DWI, DCE-MRI and texture analysis in a preclinical breast cancer model. Through the integration of multiple parameters, DWI, DCE and TexRad provide comprehensive and valuable information from biological characteristics to anatomic structures. More encouragingly, the prominent changes in key parameters before and after treatment both have good correlations and consistencies with histological results. The three imaging and analytic techniques reinforce each other and may potentially serve as non-invasive biomarkers for the guidance of treatment algorithms and in monitoring early responses to anti-angiogenesis therapy in future clinical trials.

## Abbreviations

ADC: Apparent diffusion coefficient
AUC_90_: Area under curve 90s
CER: Contrast enhancement ratio
DCE-MRI: Dynamic contrast-enhanced MRI
DMEM: Dulbecco’s minimum essential medium
DWI: Diffusion-weighted imaging
IVIM: Intravoxel incoherent motion
MPP: Mean value of positive pixels
MVD: Microvessel density
ROI: Region of interest
SD: Standard deviation
SSF: Spatial scale filter
VEGF: Vascular endothelial growth factor
VEGFR: Vascular endothelial growth factor receptor
